# Supplementary material for: VPS9D1-AS1 overexpression amplifies intratumoral TGF-β signaling and promotes tumor cell escape from CD8+ T cell killing in colorectal cancer
Source: eLife. 2022 Dec 2;11:e79811. doi: 10.7554/eLife.79811 (PMC9744440; doi:10.7554/eLife.79811)
Supplement: Figure 3—source data 1. [file elife-79811-fig3-data1.zip › Figure 3-source data 1.pptx]

## Slide 1
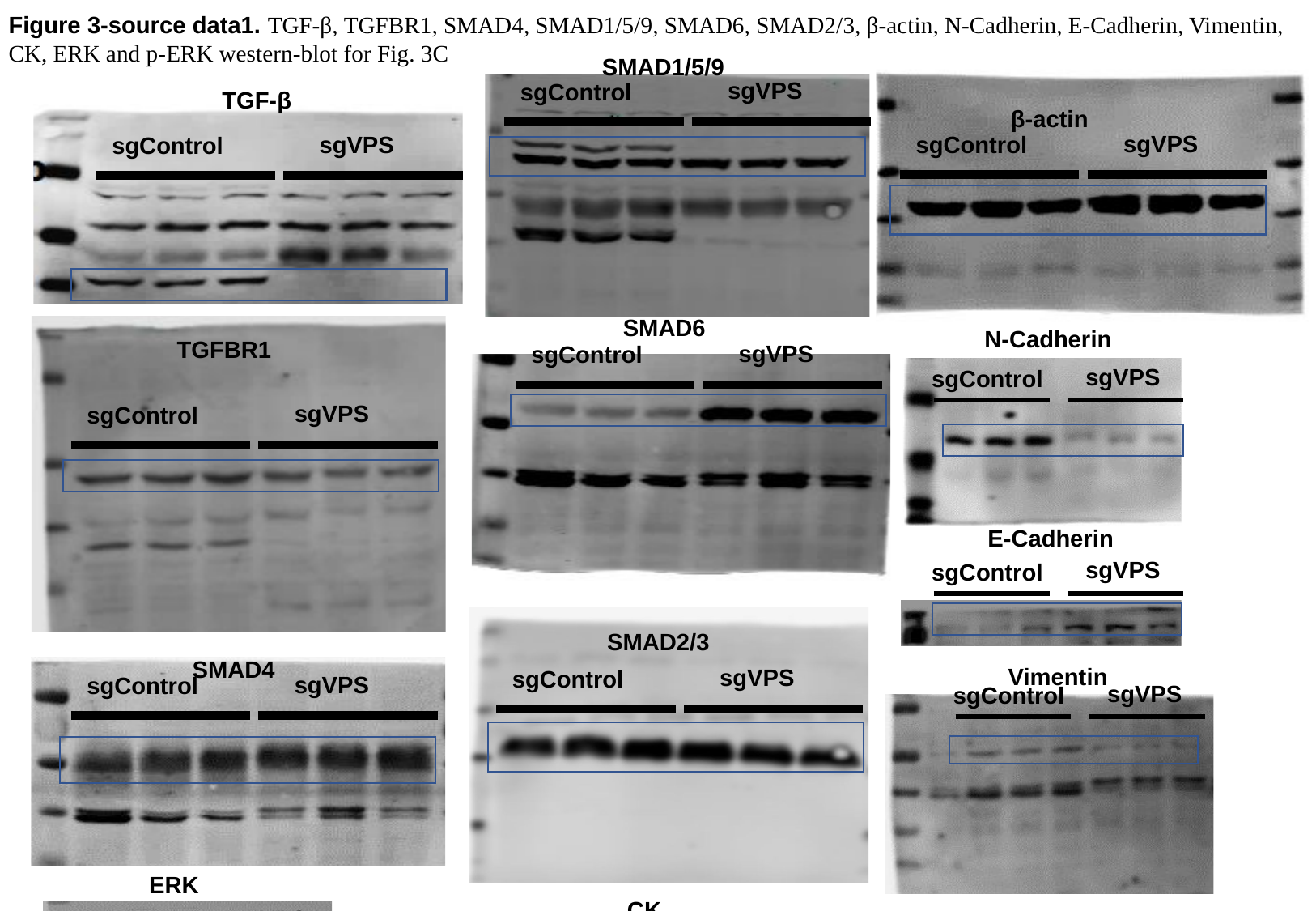

Figure 3-source data1. TGF-β, TGFBR1, SMAD4, SMAD1/5/9, SMAD6, SMAD2/3, β-actin, N-Cadherin, E-Cadherin, Vimentin, CK, ERK and p-ERK western-blot for Fig. 3C
SMAD1/5/9
TGF-β
β-actin
SMAD6
TGFBR1
SMAD2/3
SMAD4
sgVPS
sgControl
sgVPS
sgControl
sgVPS
sgControl
N-Cadherin
sgVPS
sgControl
sgVPS
sgControl
sgVPS
sgControl
E-Cadherin
sgVPS
sgControl
Vimentin
sgVPS
sgControl
sgVPS
sgControl
sgVPS
sgControl
ERK
sgVPS
sgControl
p-ERK
sgVPS
sgControl
CK
sgVPS
sgControl
