# Supplementary material for: VPS9D1-AS1 overexpression amplifies intratumoral TGF-β signaling and promotes tumor cell escape from CD8+ T cell killing in colorectal cancer
Source: eLife. 2022 Dec 2;11:e79811. doi: 10.7554/eLife.79811 (PMC9744440; doi:10.7554/eLife.79811)
Supplement: Figure 3—source data 2. [file elife-79811-fig3-data2.zip › Figure 3-source data 2.pptx]

## Slide 1
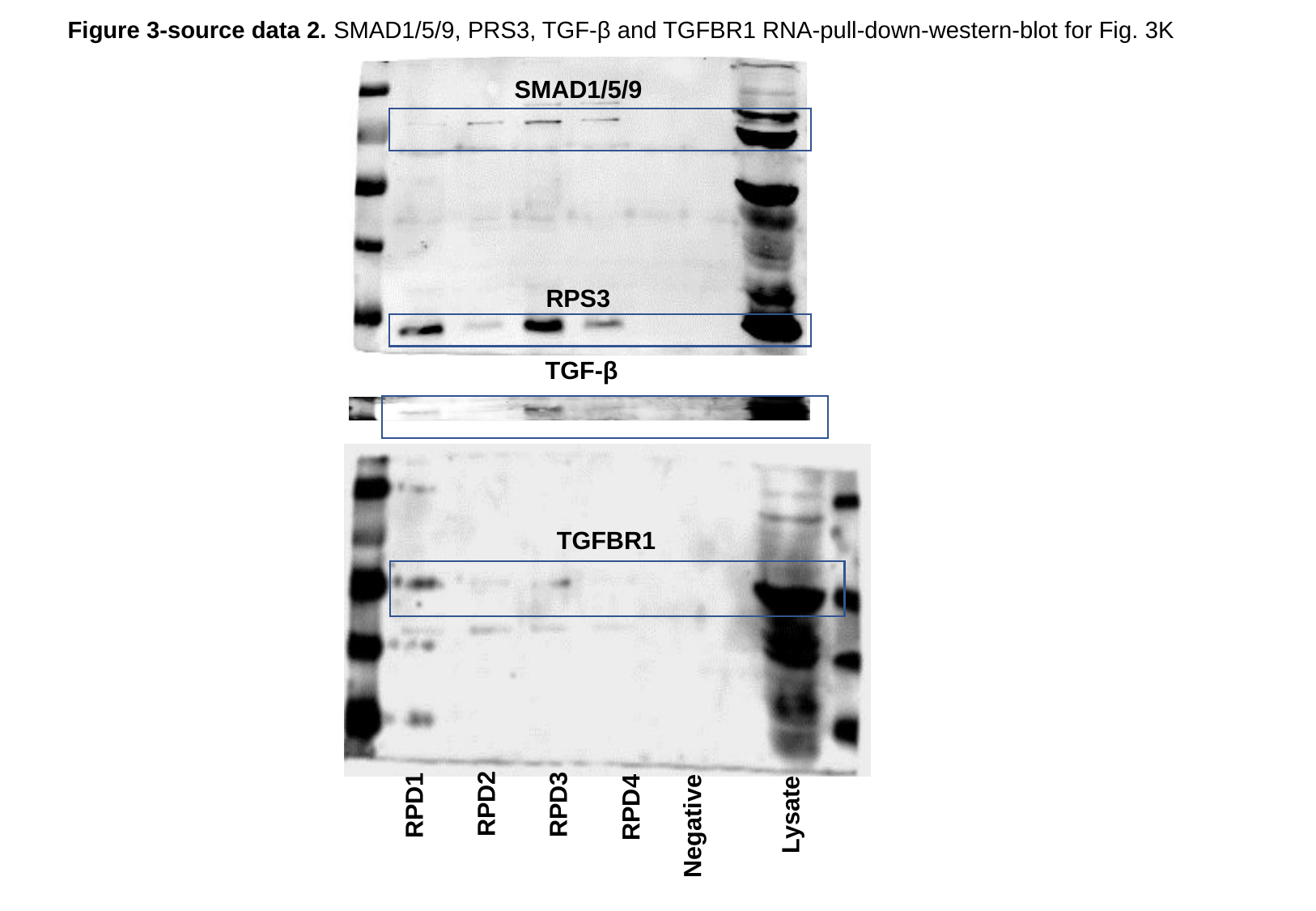

Figure 3-source data 2. SMAD1/5/9, PRS3, TGF-β and TGFBR1 RNA-pull-down-western-blot for Fig. 3K
SMAD1/5/9
TGFBR1
RPS3
TGF-β
RPD2
RPD3
RPD1
RPD4
Lysate
Negative
