# Supplementary material for: VPS9D1-AS1 overexpression amplifies intratumoral TGF-β signaling and promotes tumor cell escape from CD8+ T cell killing in colorectal cancer
Source: eLife. 2022 Dec 2;11:e79811. doi: 10.7554/eLife.79811 (PMC9744440; doi:10.7554/eLife.79811)
Supplement: Figure 3—source data 3. [file elife-79811-fig3-data3.zip › Figure 3-source data 2.pptx]

## Slide 1
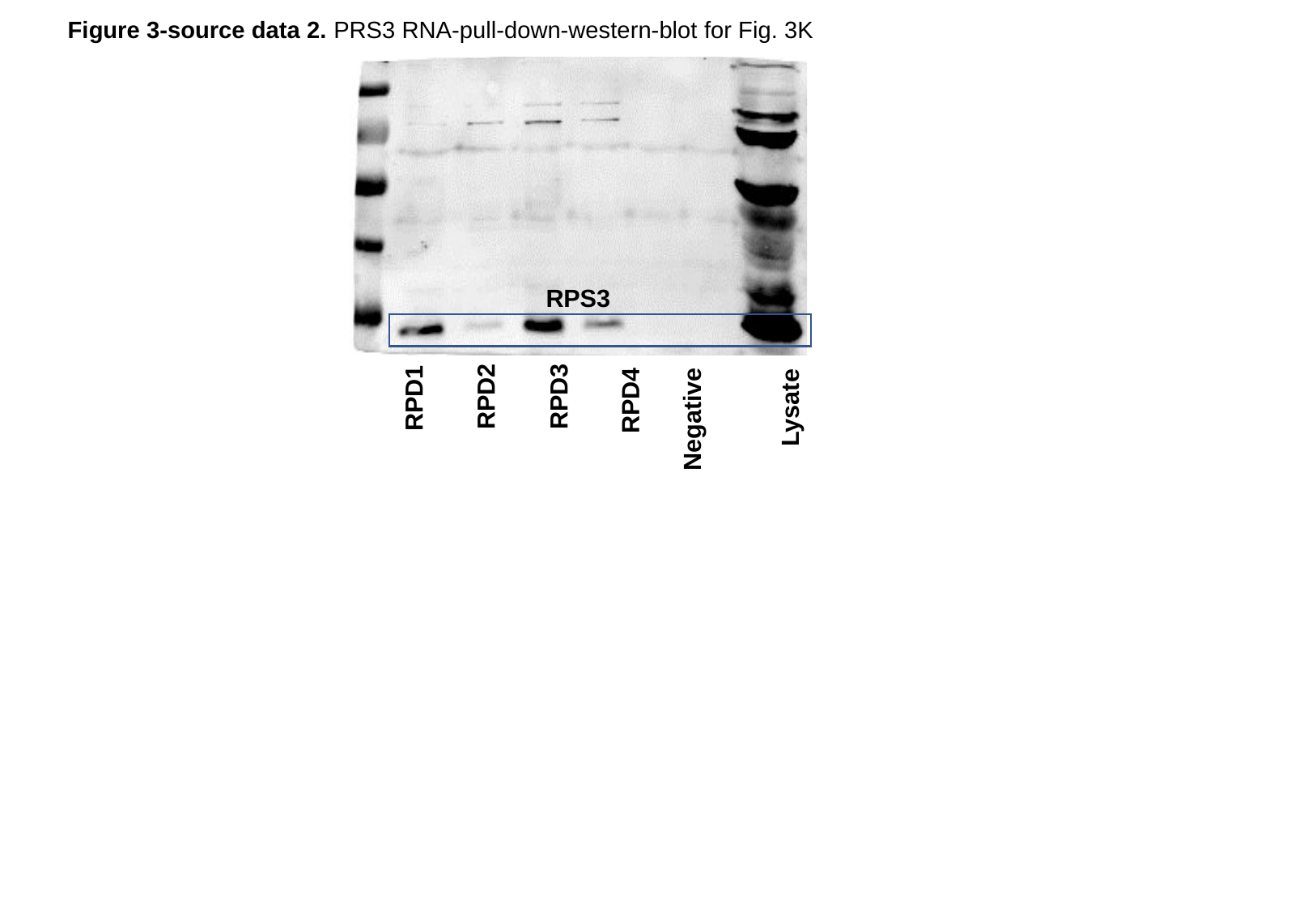

Figure 3-source data 2. PRS3 RNA-pull-down-western-blot for Fig. 3K
RPS3
RPD3
RPD2
RPD1
RPD4
Lysate
Negative
