# Supplementary material for: VPS9D1-AS1 overexpression amplifies intratumoral TGF-β signaling and promotes tumor cell escape from CD8+ T cell killing in colorectal cancer
Source: eLife. 2022 Dec 2;11:e79811. doi: 10.7554/eLife.79811 (PMC9744440; doi:10.7554/eLife.79811)
Supplement: Figure 4—source data 1. [file elife-79811-fig4-data1.zip › Figure 4-source data 1.pptx]

## Slide 1
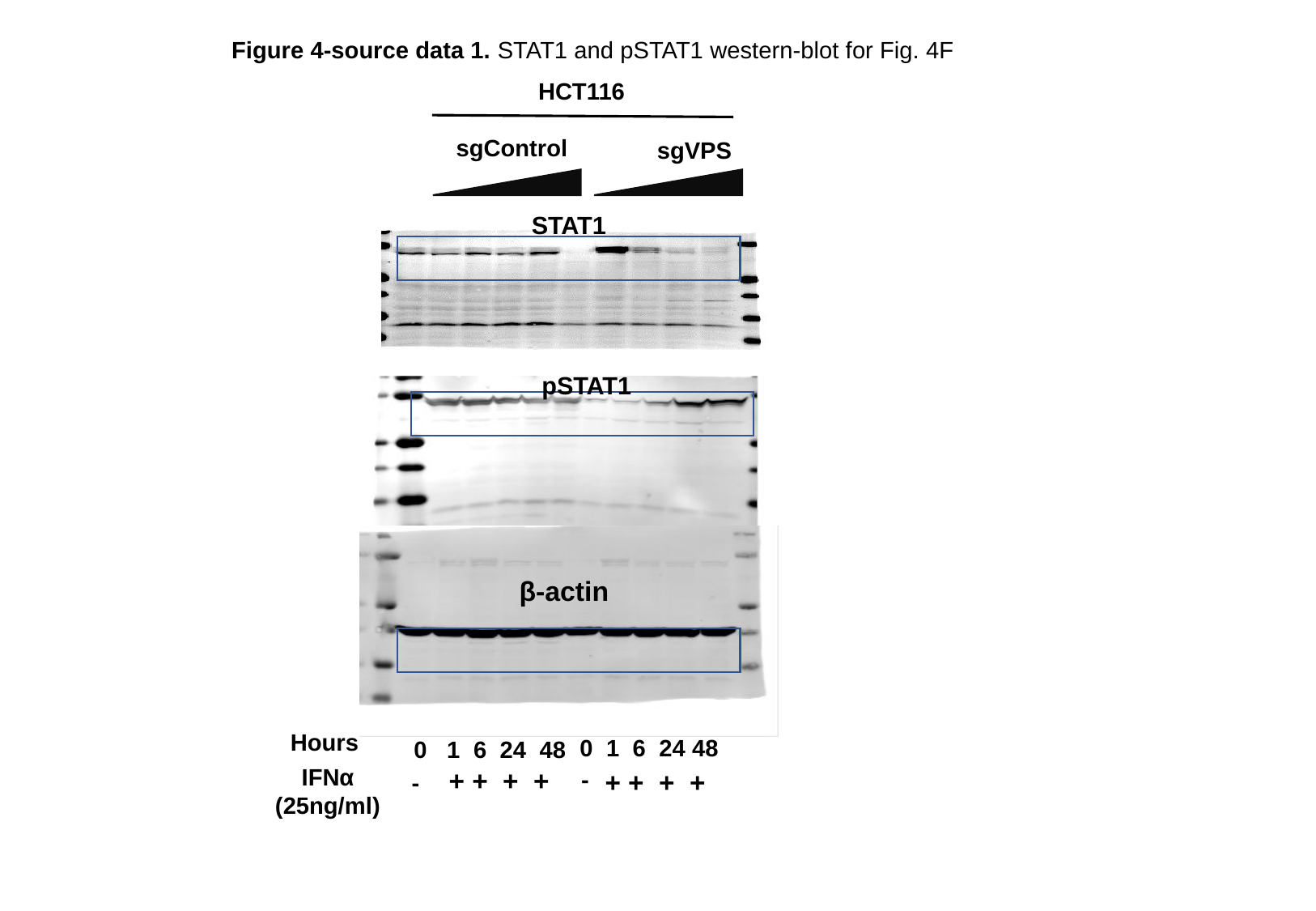

Figure 4-source data 1. STAT1 and pSTAT1 western-blot for Fig. 4F
HCT116
sgControl
sgVPS
IFNα
(25ng/ml)
0 1 6 24 48
+ + + +
+ + + +
-
Hours
0 1 6 24 48
STAT1
pSTAT1
β-actin
-
