# Supplementary material for: VPS9D1-AS1 overexpression amplifies intratumoral TGF-β signaling and promotes tumor cell escape from CD8+ T cell killing in colorectal cancer
Source: eLife. 2022 Dec 2;11:e79811. doi: 10.7554/eLife.79811 (PMC9744440; doi:10.7554/eLife.79811)
Supplement: Figure 6—source data 1. [file elife-79811-fig6-data1.zip › Figure 6-source data 1.pptx]

## Slide 1
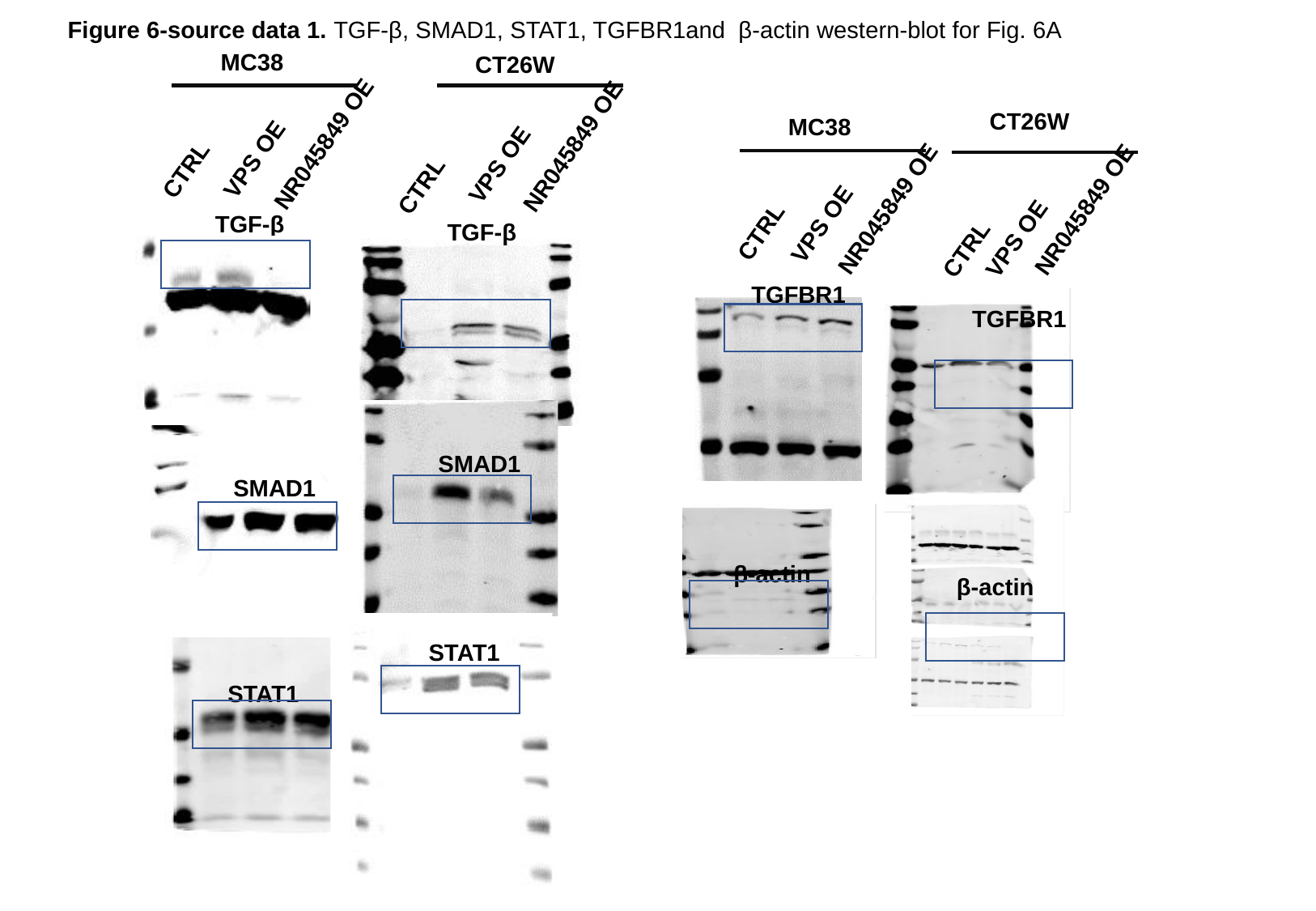

Figure 6-source data 1. TGF-β, SMAD1, STAT1, TGFBR1and β-actin western-blot for Fig. 6A
VPS OE
NR045849 OE
MC38
CTRL
VPS OE
NR045849 OE
CT26W
CTRL
VPS OE
NR045849 OE
MC38
CTRL
CT26W
NR045849 OE
VPS OE
CTRL
TGF-β
TGF-β
TGFBR1
TGFBR1
SMAD1
SMAD1
β-actin
β-actin
STAT1
STAT1
