# Supplementary material for: VPS9D1-AS1 overexpression amplifies intratumoral TGF-β signaling and promotes tumor cell escape from CD8+ T cell killing in colorectal cancer
Source: eLife. 2022 Dec 2;11:e79811. doi: 10.7554/eLife.79811 (PMC9744440; doi:10.7554/eLife.79811)
Supplement: Figure 7—source data 1. [file elife-79811-fig7-data1.zip › Figure 7-source data 1.pptx]

## Slide 1
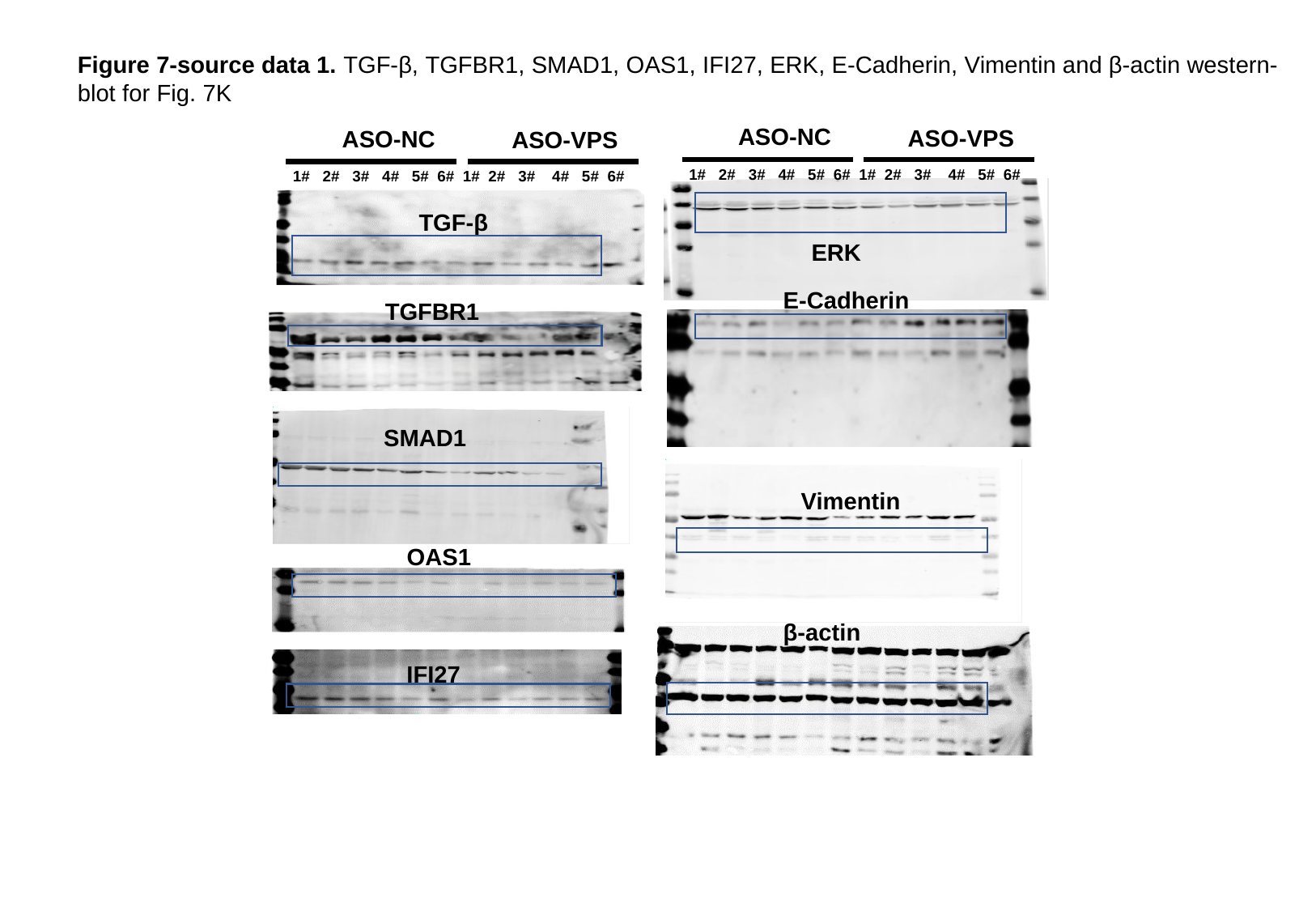

Figure 7-source data 1. TGF-β, TGFBR1, SMAD1, OAS1, IFI27, ERK, E-Cadherin, Vimentin and β-actin western-blot for Fig. 7K
ASO-NC
ASO-VPS
1# 2# 3# 4# 5# 6# 1# 2# 3# 4# 5# 6#
ASO-NC
ASO-VPS
1# 2# 3# 4# 5# 6# 1# 2# 3# 4# 5# 6#
TGF-β
ERK
E-Cadherin
TGFBR1
SMAD1
Vimentin
OAS1
β-actin
IFI27
