# Supplementary material for: VPS9D1-AS1 overexpression amplifies intratumoral TGF-β signaling and promotes tumor cell escape from CD8+ T cell killing in colorectal cancer
Source: eLife. 2022 Dec 2;11:e79811. doi: 10.7554/eLife.79811 (PMC9744440; doi:10.7554/eLife.79811)
Supplement: Supplementary file 1. — (a) sgRNA sequence. (b) Primers for PCR. (c) siRNA and shRNA sequences. (d) The primer for RNA pull down (RPD) probe synthesized. (e) The Primers for ChIP-PCR. [file elife-79811-supp1.docx]

**Supplementary Table 1a. sgRNA sequence**

|  | **Gene name** | **sgRNA** |  | **siRNA sequences (5’→3’)** |
| --- | --- | --- | --- | --- |
| 1 | VPS9D1-AS1 | VPS-sg1 | sense | CACCGACTCCAGGATGGCGGCGTAA |
|  |  |  | antisense | AAACTTACGCCGCCATCCTGGAGTC |
| 2 | VPS9D1-AS1 | VPS-sg2 | sense | CACCGCTCCAGGATGGCGGCGTAAA |
|  |  |  | antisense | AAACTTTACGCCGCCATCCTGGAGC |
| 3 | VPS9D1-AS1 | VPS-sg3 | sense | CACCGCGCCATCCTGGAGTACGAAC |
|  |  |  | antisense | AAACGTTCGTACTCCAGGATGGCGC |
| 4 | VPS9D1-AS1 | VPS-sg4 | sense | CACCGCAGGGGGACCCACTGGACGTGGG |
|  |  |  | antisense | AAACACGTCCAGTGGGTCCCCCGTC |
| 5 | OAS1 | OAS1-sg1 | sense | CACCGACAGCAGTCCAAGCTCAGTCCGG |
|  |  |  | antisense | AAACCCGGACTGAGCTTGGACTGCTGTC |
| 6 | OAS1 | OAS1-sg2 | sense | CACCGGTACTGAGTTCGCTCCAGCT TGG |
|  |  |  | antisense | AAACCCAAGCTGGAGCGAACTCAGTACC |
| 7 | OAS1 | OAS1-sg3 | sense | CACCGGCAGCTGGCACAAGAGGCTGAGG |
|  |  |  | antisense | AAACCCTCAGCCTCTTGTCCAGCTGCC |

**Supplementary Table 1b. Primers for PCR**

|  | **Genes** |  | **Primer sequences (5’→3’)** |
| --- | --- | --- | --- |
| VPS9D1-AS1 Norther blot PCR primers | | | |
|  |  | sense | 5'-TTGGCTTCAGGCGTGTTT-3' |
|  |  | antisense | 5’- AGATTGAGGAGTTGGGTGGA-3’ |
| qRT-PCR Primers | | | |
| 1 | VPS9D1-AS1 | sense | TGCTAGCTTCAGCATCTTGG |
|  |  | antisense | AAGTAACAGTGGTAGAGCCGA |
| 2 | HLA-DRA | sense | ATGGTCAGACTCTATTACACCCCA |
|  |  | antisense | GTCAGTAGAGCTCGGGAGTG |
| 3 | IRF7 | sense | GACGTCGCCTTTCCCGTC |
|  |  | antisense | GTGTTGAACCAGTGTCCAGG |
| 4 | MX1 | sense | CGAGCAGAAATGAAACCGAAA |
|  |  | antisense | TTACTTCTCCCTGCGCTCTC |
| 5 | SP100 | sense | GAAGCAAGCATGCTCCTATGAC |
|  |  | antisense | AAACGTCTCTTCCTGCTGGG |
| 6 | BST2 | sense | CCAGGCCAGACTCCTTTCAG |
|  |  | antisense | CTTACAGCGCTTATCCCCGT |
| 7 | ICAM1 | sense | ATTTGTTCCGGAGGGGAAGG |
|  |  | antisense | GCGCGTGATCCTTTATAGCG |
| 8 | ISG15 | sense | CTGTTTAGGTTTCGCTTTCCCG |
|  |  | antisense | GTGTTGAACCAGTGTCCAGG |
| 9 | PTAFR | sense | AGAAGCCGTCCAGGAAACATGCTC |
|  |  | antisense | GTGTGTCTCTGTCTGGGTCCTG |
| 10 | HERC5 | sense | GGAAAATGACTGTGGACGCT |
|  |  | antisense | TCCTCAATTGCTGCCGACC |
| 11 | HLA-DPA | sense | CTAGAGGCCCACAGTTTCAGT |
|  |  | antisense | GTCTTCAGGGCGCATGTTGT |
| 12 | HLA-F | sense | GCAGAGCAGTCTCCCCAG |
|  |  | antisense | GTCCCACACAAGGAAGCTGT |
| 13 | IFI6 | sense | CTCCAAGGTCTAGTGACGGA |
|  |  | antisense | TTCTTACCTGCATCCTTACCC |
| 14 | IFI27 | sense | GCCTCTGCTCTCACCTCATC |
|  |  | antisense | ACAGCCACAACTCCTCCAAT |
| 15 | IFITM3 | sense | AAGGAAACTGTTGAGAAACCGA |
|  |  | antisense | TCATGGTGTCCAGCGAAGAC |
| 16 | OAS1 | sense | GTGAGCTCCTGGATTCTGCT |
|  |  | antisense | GGCCTTTGGCAAGAGGTAAGT |
| 17 | OAS3 | sense | CCGGGCGGGAAAACGAAAC |
|  |  | antisense | GCCTTCTCTACGAACTCCTTCC |
| 18 | STAT1 | sense | AACCTCGACAGTCTTGGCAC |
|  |  | antisense | CACTGAGACATCCTGCCACC |
| 19 | 18S | sense | AAACGGCTACCACATCCA |
|  |  | antisense | CACCAGACTTGCCCTCCA |
| 20 | TGFB1 | sense | ATGGAGAGAGGACTGCGGAT |
|  |  | antisense | TAGTGTTCCCCACTGGTCCC |
| 21 | TGFBR1 | sense | TCCAACTACTGTAAAGTCATCACC |
|  |  | antisense | GGGTCCTCTTCATTTGGCAC |
| 22 | SMAD1 | sense | GGAGAAAGGAGAGGCCGAG |
|  |  | antisense | AAAAAGTAACCCAGTCAGCACCG |
| 23 | SMAD5 | sense | AATCTGCCTCTGACTTGACCC |
|  |  | antisense | CGGAGACCTTCCTGTAACTCAA |
| 24 | SMAD9 | sense | AGTGGCCAACCTGTAGATGC |
|  |  | antisense | GAAGCCTGGAATGTCTCCCC |

**Supplementary Table 1c. siRNA and shRNA sequences**

|  | **Genes** |  | **siRNA sequences (5’→3’)** |
| --- | --- | --- | --- |
| 1 | TGF-β | sense | ACAACGAAAUCUAUGACAATT |
|  |  | antisense | UUGUCAUAGAUUUCGUUGUTT |
| 2 | TGFBR1 | sense | GAACAGAAGUUAAGGCCAATT |
|  |  | antisense | UUGGCCUUAACUUCUGUUCTT |
| 3 | SMAD1 | sense | GGAUAAAGUUCUUACUCAATT |
|  |  | antisense | UUGGUAAGAACUUUAUCCTT’ |
| 4 | SMAD5 | sense | GAGCUAAAGCCGUUGGAUATT |
|  |  | antisense | UAUCCAACGGCUUUAGCUCTT |
| 5 | SMAD9 | sense | GCAAGGAGAUGAAGAGGAATT |
|  |  | antisense | UUCCUCUUCAUCUCCUUGCTT |
| 6 | Negative control siRNA | sense | UUCUCCGAACGUGUCACGUTT’ |
|  |  | antisense | ACGUGACACGUUCGGAGAATT |
| 17 | IFNAR1-shRNA | -1 | GCCAAGATTCAGGAAATTATT |
|  |  | -2 | CCTTAGTGATTCATTCCATAT |
|  |  | -3 | GCTCTCCCGTTTGTCATTTAT |

**Supplementary Table 1d. The Primer for RNA Pull Down (RPD) probe synthesized**

|  | **Probes** |  | **Primer sequences (5’→3’)** |
| --- | --- | --- | --- |
| 1 | RPD1 | sense | TGACCTGTGTGATCCTACGTGCCGAGCGT |
|  |  | antisense | TCACATTTCCAGAGAGCTGACG |
| 2 | RPD2 | sense | TGACCTGTGTGATCCTACGTCATCTTGGAG |
|  |  | antisense | AAGTAACAGTGGTAGAGCCGAC |
| 3 | RPD3 | sense | TGACCTGTGTGATCCTACGTCTGCTGCCCTTC |
|  |  | antisense | TCAGGGGGAGCTAACAGTTG |
| 4 | RPD4 | sense | TGACCTGTGTGATCCTACGTCGATTTAGCCC |
|  |  | antisense | CTCTGGCGCTCCAAGATTGA |

**Supplementary Table 1e. The Primers for ChIP-PCR**

|  | **Genes** |  | **Primer sequence (5’→3’)** |
| --- | --- | --- | --- |
| 1 | OAS1 ChIP1 | sense | ACGTTTATAGAGGCAATTTTGTAGT |
|  |  | antisense | AACCTCCAGCCAACATAAATCT |
| 2 | OAS1 ChIP2 | sense | CCACCCTCATGGTGACATTTAGT- |
|  |  | antisense | AACCAGATTATTCATGAGTTGACAC |
| 3 | OAS1 ChIP3 | sense | AGTTCAGAGAAAGGCTGGGC |
|  |  | antisense | CCTCGGAAGCACCTTTCCTT |
| 4 | IFI27 ChIP1 | sense | GCGTAGAGCACACTCCCATC |
|  |  | antisense | CCAAAGGGTGTGATCCCAGG |
| 5 | IFI27 ChIP2 | sense | CGGAACATCTGCCTATCGCA |
|  |  | antisense | AGGATTTGCCCATGGCATCA |
| 6 | IFI27 ChIP3 | sense | ATATGGACCAGGGTGGCCTT |
|  |  | antisense | TCAGAACAACTTGCATAACAGCC |
